# Age-Based Differences in Care Setting Transitions over the Last Year of Life

**DOI:** 10.1155/2011/101276

**Published:** 2011-08-07

**Authors:** Donna M. Wilson, Jessica A. Hewitt, Roger Thomas, Deepthi Mohankumar, Katharina Kovacs Burns

**Affiliations:** ^1^Faculty of Nursing, University of Alberta, Edmonton, AB, T6G 2G3, Canada; ^2^Department of Family Medicine, Faculty of Medicine, University of Calgary, Health Sciences Centre, 3330 Hospital Drive NW, Calgary, AB, T2N 4N1, Canada; ^3^Health Sciences Council, University of Alberta, Edmonton, AB, T6G 1K8, Canada

## Abstract

*Context*. Little is known about the number and types of moves made in the last year of life to obtain healthcare and end-of-life support, with older adults more vulnerable to care setting transition issues. *Research Objective*. Compare care setting transitions across older (65+ years) and younger individuals. *Design*. Secondary analyses of provincial hospital and ambulatory database data. Every individual who lived in the province for one year prior to death from April 1, 2005 through March 31, 2007 was retained (*N* = 19, 397). *Results*. Transitions averaged 3.5, with 3.9 and 3.4 for younger and older persons, respectively. Older persons also had fewer ER and ambulatory visits, fewer procedures performed in the last year of life, but longer inpatient stays (42.7 days versus 36.2 for younger persons). *Conclusion*. Younger and older persons differ somewhat in the number and type of end-of-life care setting transitions, a matter for continuing research and healthcare policy.

## 1. Introduction

Rapid population aging is occurring now in most developed and developing countries, leading to an increased interest in palliative and end-of-life care [1, 2]. Dying people are often older, age 65 or more [3], and for terminally ill individuals and especially older persons, smooth transitions from acute cure-oriented care to palliative care and from one care setting to another are essential for quality of life remaining [4–7]. Care setting relocations are typically considered the moves that an individual makes from one place to another to obtain healthcare and other supports needed to address their end-of-life care needs, with the person's home often the main end-of-life care setting [8]. Each move, however, requires a number of transitions, such as in care providers, aims of care, technologies available for use, and other less tangible factors such as fatigue, pain, and frustration with having to move and/or relief with moving to a care setting where current care needs can be met. End-of-life care setting transitions are therefore more than just a physical move from one care setting to another; they are also the physical, psychological, emotional, and spiritual changes and impacts that occur as a result of the temporary or permanent moves made in the year before death to obtain healthcare and other needed supports. To date, few studies have focused on the number and types of moves that a person makes in the last year of life to obtain healthcare and other end-of-life support, an issue as this information would assist planning appropriate end-of-life health policy and care and help to avoid unnecessary or difficult care setting transitions that are traumatizing for the individual and their family [9].

## 2. Background

As indicated, few studies have focused on how often people move during the last year of life, although many studies have indicated that hospital utilization tends to be high in the last year of life [10]. Few of these studies have considered the impact of having to move to receive healthcare and other needed end-of-life services in the last year of life. Some difficulties associated with care setting transitions near death have been studied, such as burden to caregivers and cost of hospital transfers [11–14]. Hospital readmission has also been a focus of some research, with evidence now that 12–25% of hospital discharges in the last year of life are followed by a hospital readmission, and with almost 50% of these readmissions through an emergency room or ER [15, 16]. 

Older frail and older terminally ill individuals are particularly vulnerable to difficult care setting transitions. Older individuals have a higher prevalence of chronic and terminal illnesses that require general and specialist medical attention, and so they tend to frequently visit a wide range of healthcare practitioners [17, 18]. Healthcare services today are most often provided on a day surgery or outpatient basis, instead of in hospital after admission there, with older persons thus at risk of needing to make frequent same-day trips to obtain health care and then return home. Healthcare technologies and high-tech hospital services have also become centralized in larger cities, with all persons not living in larger cities needing to travel to access these services [19]. Traveling long distances when ill is understandably difficult, and this difficulty is increased when the person is terminally ill. In Canada, specialized palliative care services have remained centralized in larger cities and in larger hospitals, so accessing palliative care services may also involve considerable travel time and additional complexity associated with moving terminally ill people from one place to another [20, 21]. 

Each trip to obtain needed healthcare services or other supports means a change in care setting. Moves or transfers from one care setting to another often result in care gaps or issues, such as discontinuity in care planning. A recent study found hospital discharge summaries were available for only 12–34% of repeat office or hospital visits, with this gap identified as leading to poor quality of care in 25% of all cases [22]. Other issues, such as increased risk of medical error, are also of concern with care setting changes. Around 50% of medication errors are thought to occur during care setting transitions [23]. For older adults, errors in clinical plans and medications are often more harmful as they are less resilient and more vulnerable to serious illnesses than younger persons [24–26]. The importance of minimizing the number of care setting transitions when terminally ill to reduce any negative impacts or effects of moving cannot be emphasized enough.

Care setting transitions of any kind can be a psychological burden for older adults, in large part because of the stress of leaving a familiar environment and familiar people, often their own home and family members or friends [27–29]. For a terminally ill person, every departure from home to hospital or another care setting could be considered a major emotional risk, as they must realize they may never return home again. Travelling long distances to see specialists or have diagnostic tests performed to diagnose progressive disease poses additional risks and burdens, with older persons potentially much more impacted than younger persons. Elderly individuals are more likely to have complicated or difficult and lengthy hospitalizations prior to being discharged home or to a nursing home for continuing care [30]. In short, although care setting transitions may be necessary, they can cause psychological, economic, physical, and social burdens; burdens that are more commonly impactful on older adults. It is therefore important to determine the number and types of end-of-life care setting transitions across older and younger persons so as to gain evidence for health policy and healthcare services planning.

## 3. Methods

The paucity of research on the number and types of end-of-life care setting transitions and concern for older terminally ill persons who are more at risk from care setting transitions provided the impetus for a research study. This study involved secondary analyses of complete population-level hospital and ambulatory care data to examine care setting transitions in the last year of life and determine if there were differences in the numbers and types of care setting transitions for older versus younger individuals who had lived one full year in Alberta, a Canadian province, at any time from April 1, 2005 through March 31, 2007. 

### 3.1. Data and Participants

Complete individual anonymous data for two recent years were obtained from Alberta Health and Wellness upon request. The data received were individual anonymous data on all persons in the province's healthcare registry (sociodemographic data), inpatient hospital, and ambulatory care (ER, outpatient clinic, and day surgery clinic) databases. Alberta Health and Wellness is a government agency that collects and then supplies healthcare data to researchers. Research ethics approval is required prior to data delivery, with the University of Alberta's Health Research Ethics Board supplying this approval. A total of 19,397 persons who had died in Alberta in the 2006-07 year had one full year of data before death available for analysis. In total, 3,216,624 care episodes were attributed to these 19,397 individuals.

Data cleaning and manipulation using ACCESS were first required to ensure that all data for analysis were error free (such as 999 recorded as an age instead of as missing data) and that the data reflected only those individuals who had lived for 365 days in Alberta prior to death in Alberta. In addition, care was taken to ensure that all data linkages across the three databases were correct for each subject and that the compiled data were comparable across subjects, with a composite database constructed for this purpose. The composite database data were then analyzed using the SPSS computer program (version 18). As indicated, analyses were restricted to individuals who had at least one complete year of information, which excluded children under the age of 1, any persons who died shortly after moving to the province, and any persons who died out of province. Each care setting transition was defined as any move made in the last 365 days of life, as identified and tabulated from the data contained in the original databases. The composite database thus contained information on every care setting transition, which could be a move from home to hospital for inpatient admission or to visit an ER or ambulatory care clinic, a move required by a discharge home from hospital or an ER or ambulatory care clinic, a transfer from one hospital to another, a transfer from hospital to nursing home, or nursing home to hospital. In addition, the composite database contained data for many other variables per subject, including their total number of inpatient hospital days accumulated in the last year of life, with this total number of stay days calculated by adding all days for each hospitalization episode. Understandably, not all subjects were hospitalized for inpatient care in the last year of life, and not all subjects visited ERs or ambulatory care clinics in the last year of life.

### 3.2. Data Analyses

The main focus of analyses was to determine if there were differences between younger persons and older adults (age 65+) in the number and type of care setting transitions. To meet this goal, two sets of analyses were conducted. First, descriptive and exploratory analyses were conducted to determine the number of care setting transitions, length of each hospital stay, total inpatient hospital stay days, number of visits to all types (provincial, regional, or local) of hospitals, number of surgical and other procedures performed in the last year of life, number of palliative care visits or admissions, number of cancer care visits or admissions, and also the number of visits to ambulatory care clinics (i.e., outpatient and/or day surgery clinics combined). Sociodemographic variables, such as gender and rural/urban status, were also examined and compared across younger/older subjects. Counts, percentages, chi square, and *t*-tests were computed to describe the above variables for all subjects collectively and then younger persons and older adults separately. *t*-tests for independent samples were used to determine if there were significant age-based differences in the number of care setting transitions in the last year of life. 

Logistic regression analysis was then performed to assess care setting transition and healthcare utilization differences between younger and older subjects, with complete information on 10,897 subjects available for this analysis. Gender and urban/rural status were initially included as covariates but were removed because they did not improve model fit. The variables included in the logistic regression model ultimately were total inpatient stay days; then as a second set of covariates, number of diagnoses, number of all procedures received in the hospital setting, number of surgical procedures specifically; then as a third set the number of care setting transitions, and as the final set the number of outpatient visits and day surgery visits, number of outpatient and day surgery procedures, number of ER visits, and number of ER procedures.

## 4. Results

### 4.1. Initial Sociodemographic and Care Setting Transition Findings

Nearly 3/4 (73%) of the 19,397 subjects were 65 years of age or older (*n* = 14,168), with a slight preponderance of all subjects being male (*n* = 10,008, 51.6%). More of the younger subjects were male (60.1%, *n* = 3,145), while slightly more of the older subjects were female (51.6%, *n* = 7,306). The majority (81.6%) were urban dwellers (including 79.6% of those <65 and 82.6%  ≥65).

The 19,397 subjects averaged 3.5 care setting transitions in the last year of life (range of 1–41, standard deviation = 3). A large proportion (81.0%) had between 1 and 5 care setting transitions, while only 3.3% (*n* = 454) had more than 10 transitions. Total inpatient days averaged 41 for all subjects combined. Two-thirds (68.0%) had 1–5 inpatient hospital separations, with the remaining often having no hospital separations, but a small minority (2.1%) had 6 or more admissions. Most individual hospitalizations were greater than 10 days for 72.8% of the subjects, but 15.2% had stays of only 1–5 days. The length of stay for the 9,270 persons who were admitted to a very large provincial hospital was typically only 1–5 days in length, with only 0.1% staying more than 10 days. A majority (84.3%) had no admission to any of the medium-sized regional hospitals in mid-sized cities or small (local) hospitals in towns or small cities. Only 15.4% of all subjects were admitted to a regional hospital, with stays there almost always 1–5 days in length. 

The 19,397 subjects had an average of 2.4 major diagnostic or treatment procedures performed on them during their last year of life. Almost half (49.1%) had 1 to 5 procedures performed, with the remaining almost equally split into those who had none and those who had more than 5 procedures performed. In addition, 43.7% (*n* = 6,063) had undergone one or more surgical procedures in the last year of life. Many of these procedures were performed in ambulatory care settings. Total visits to outpatient and day surgery clinics ranged from 1 to 5 times for nearly half of all subjects (47.0%, *n* = 9,112), and 66.4% of all subjects (*n* = 12,876) were admitted to an ER 1 to 5 times. Only 27.4% of all subjects (*n* = 5,314) had one or more palliative care hospital admissions or ambulatory care visits, with 21.6% having accessed hospitals or ambulatory care settings to receive cancer care.

### 4.2. Comparisons

As indicated above, care setting transitions averaged 3.5 across all subjects, but with 3.9 and 3.4 transitions for younger and older persons, respectively. As shown in Table 1, this difference was significant [*t* = 8.3, *P* < .05]. In contrast, younger and older subjects did not differ significantly in the number of inpatient hospital separations [*t* = 0.48, *P* > .05; as $\overset{̅}{X}=1.6$ for younger and older subjects]. However, some differences were present as only 62.0% of younger subjects as compared to 70.2% for those aged ≥65 had 1 to 5 inpatient hospital separations. Younger and older subjects differed significantly in the total number of inpatient days of care accumulated over the year, with older persons hospitalized more days on average [*t* = 9.9,*P* < .05, $\overset{̅}{X}=36.2$ younger subjects versus $\overset{̅}{X}=42.7$ for older subjects]. Regardless, the majority (75.4%) of older subjects and the majority (65.2%) of younger subjects had individual hospital stays over 10 days in length. Older subjects had longer stays in large provincial hospitals as compared to younger subjects ($\overset{̅}{X}=32.4$ younger versus $\overset{̅}{X}=39.8$ older), a nonsignificant difference. Older subjects also had longer stays in regional and local hospitals as compared to younger subjects (Means of 28.4 and 34.5 versus 24.9 and 24.9 for older and younger subjects, resp.), another nonsignificant difference.

In contrast, younger subjects had a greater number of procedures (total and surgical only) performed on them in comparison to older subjects (Means of 3.6 and 2.6 for younger subjects versus 2.0 and 1.3 for older subjects, resp.). These differences were statistically significant (*t* = 22.2, *P* < .05 for total procedures and *t* = 23.3, *P* < .05 for surgical procedures only). In addition, younger subjects had a significantly higher average number of visits to outpatient clinics ($\overset{̅}{X}=6.2$ younger versus $\overset{̅}{X}=4.6$ older) and a significantly higher average number of visits to ERs ($\overset{̅}{X}=3.2$ younger versus $\overset{̅}{X}=2.7$ older) (*t* = 19.6, *P* < .05 for outpatient clinics and *t* = 16.2, *P* < .05 for ER). The number of procedures performed at outpatient clinics ($\overset{̅}{X}=12.0$ younger versus $\overset{̅}{X}=8.7$ older) and in ERs ($\overset{̅}{X}=4.1$ younger versus $\overset{̅}{X}=3.6$ older) was also higher for younger subjects (*t* = 27.8, *P* < .05 for outpatient procedures and *t* = 8.8, *P* < .05 for ER procedures, resp.). However, younger and older subjects had a similar number of day surgery visits ($\overset{̅}{X}=2.9$ younger versus $\overset{̅}{X}=2.4$ older) and day surgery procedures ($\overset{̅}{X}=17.5$ younger versus $\overset{̅}{X}=17.4$ older); both were nonsignificant differences. In addition, younger and older subjects had a similar (nonsignificant) number of visits for palliative care ($\overset{̅}{X}=1.3$ younger versus $\overset{̅}{X}=1.1$ older) and a similar (nonsignificant) number of visits for cancer care ($\overset{̅}{X}=1.9$ younger versus $\overset{̅}{X}=1.6$ older).

### 4.3. Logistic Regression Findings

The above identified differences between older and younger subjects were emphasized by the findings of the logistic regression analysis. Model fit was determined as needing to remain significant when variables were entered. This indicates that the model with variables of interest is a better fit for the data over the null model. The model with total number of inpatient stay days added in was significantly different from the constant only model (see Table 2 model summary). Older subjects had 1.01 greater odds of longer inpatient stays as compared to younger subjects (*χ* ^2^(1) = 10.9, *P* < .05). The overall model continued to remain significant with the addition of number of diagnoses, number of procedures, and number of surgical procedures (*χ* ^2^  (4) = 521.1, *P* < .05). The addition of the number of care setting transitions also did not change model fit, with the odds ratio of .881 indicating a small but still significant difference between younger and older subjects (*χ* ^2^(5) = 669.3, *P* < .05). The final model, including the number of visits to outpatient clinics, number of procedures in outpatient clinics, number of ER visits, and number of procedures in emergency rooms continued to remain significant (*χ* ^2^(9) = 785.9, *P* < .05). In the final model, older subjects had 1.01 higher odds of longer inpatient stays compared to younger subjects. Older subjects also had .92 lesser odds of greater total number of procedures and .91 lesser odds of more care setting transitions than younger subjects. 

## 5. Discussion

The subjects in this end-of-life care setting transitions study were mainly older adults, aged 65 years or older, a finding that is consistent with previous age-based findings in other end-of-life studies [3, 9]. Although younger subjects were more often male, there was a slightly larger number of women among the older subjects, with this finding expected as females tend to live longer than males [31]. The average number of care setting transitions was only 3.5 for all subjects, which is not a large number and could simply indicate two trips to a hospital, a hospital ER, or another ambulatory care setting. A small proportion (4%) had more than 10 care setting transitions in their last year of life. Contrary to expectations, persons under the age of 65 had a significantly higher average number of care setting transitions in the last year of life. This is a major finding, as older people are typically considered high users of healthcare services as they near death [10]. It is possible that death is a more expected outcome of illnesses occurring in old age as compared to illnesses occurring among persons who are less than 65 years of age, with visits to healthcare facilities for diagnostic and treatment efforts thus understandably differing. It is also possible that the illnesses suffered by younger people and older people differ in type and severity, such that younger people are more in need to healthcare and other supports over the last year of life. 

Although the average number of transitions for all subjects (3.5) and across older and younger subjects (3.4 versus 3.9, resp.) were relatively low, it is also important to note that 4% had 10 or more care settings transitions, with 41 the highest number of care setting transitions. Some persons clearly travelled more often to access healthcare and other end-of-life supports, and each of these trips could involve many hours of travel. Although these persons and all of the others would have likely benefitted overall from this travelling to access health care and other end-of-life supports, it could also be argued that any and all care setting transitions occurring in the last year of life represent a large number of risks and other considerations or adjustments to be made by the individual and their family. In addition, healthcare workers must adapt to a patient who could vary considerably in care needs from one time to another, as care needs typically vary over the course of terminal illnesses. While 3.5 moves or care setting changes may not appear to be burdensome, each care setting transition should be optimized so that high quality care is obtained upon arrival and that the move from one place to another is optimized as much as possible. For instance, long stays in ERs prior to admission to hospital could and should be minimized for persons designated as terminally ill. In some cases, care should be taken to reduce the number of care setting transitions as each poses risks and burdens regardless of the potential benefit. 

It is also remarkable that over the last year of life, people under the age of 65 had a higher average number of total procedures performed, a higher average number of surgical procedures performed, a higher average number of procedures performed in the ER, and a higher average number of procedures performed in outpatient clinics as compared to persons 65 years of age or older. There may be some important reasons for these age-based differences. One reason could be the tendency to provide cure-oriented care for younger individuals and the corresponding tendency to more often provide noncurative or palliative care for older individuals who are less likely to survive aggressive curative treatments such as major surgery and chemotherapy. Although younger persons may benefit from aggressive curative treatment by surviving, younger irrevocably dying individuals could be subject to more futile care in the last year of life, a major concern. This concern brings to attention the importance of advance care planning for people of all ages [6, 32]. In addition, with improved diagnostic tests, it is becoming more and more obvious when an illness is incurable and also when dying is becoming more immediately evident. Both younger and older persons should be able to benefit from these prognostication advancements. 

Ageism is another possible concern with the higher procedure rates among younger versus older persons. Age-based discrimination could be actively or passively occurring, and this is highly problematic if older people who could potentially benefit from diagnostic and therapeutic procedures are not offered them. The finding that older subjects had a higher number of total days in hospital in the last year of life could simply be an outcome of their having received less diagnostic or treatment-oriented healthcare services previously or their having less timely access to needed healthcare services. Prolonged stays in hospital could also be a factor of the greater difficulty in moving older persons from one place to another. Older subjects with rural residences in particular would have long travelling distances to access healthcare and other end-of-life services, as rural areas typically have minimal healthcare services overall [33, 34]. Travelling from rural areas to urban areas and from one urban area to another could also be highly problematic for older people if family members and friends are not able or available for these moves. Difficulties in travel could mean that older people are at risk from refusing tests and treatments that could be beneficial to them. The longer inpatient hospital stays for older subjects are also explained by a higher incidence of chronic illnesses and disabilities with aging, health conditions that often necessitate longer hospital stays as recovery is more complicated [17, 30]. 

Although longer hospital stays may be indicated for older terminally ill persons with both acute and additional underlying health reasons, the impact of long hospital stays on terminally ill or dying individuals of all ages and their families must be considered. The majority of individuals in both age groups typically had hospital stays of 10 days or more. Respite for family caregivers could be a welcome benefit to both the family caregiver and care recipient, but separations from home and family can have serious consequences. One of the greatest concerns is that death can suddenly take place in hospital, with family and friends not present. Sudden death and other sudden health crises, in the absence of a living will, could also result in life support being initiated even when this intensive care is expected to have a negative or nominal outcome. 

This study also showed that only about 1 in 5 subjects received specialized palliative care. It is important to note that in Alberta few regional and local hospitals have palliative care specialists and specialist palliative care services, with some trips to large provincial hospitals thus likely made specifically for specialist palliative care. This gap in basic hospital services is highly problematic, as most individuals nearing the end of life could benefit from specialized palliative care. This is not the first study that identified gaps in palliative care services [26]. In Alberta, as there are only two cities with large provincial hospitals, most terminally ill persons could have considerable travelling distances to access specialist palliative care services. This travelling is typically by private car and through family drivers. With seasonal weather, these trips to and from hospitals could be very burdensome for both the terminally ill person and their family. Expanding specialist palliative care services to regional and local hospitals would have the advantage of ensuring that more people overall would be able to access palliative care and access it more easily as well.

## 6. Conclusion

Healthcare age disparities have been a concern for some time, with older people more often assumed to be high users of hospitals and other healthcare services in the last year of life. The findings of this study revealed that younger people are more often admitted to ERs and outpatients clinics, and thus they have a significantly higher number of care setting transitions in the last year of life as compared to older persons. Some additional health services utilization differences were apparent, such as a higher total inpatient care days for older persons. The nature of these care setting transitions and their impact on dying individuals and their families need to be further examined for quality of life and quality of care considerations. One concern is that older dying persons are not able to return home to be in familiar surroundings and with familiar family caregivers but instead are retained in hospital. All end-of-life care setting transitions, particularly if they are well above the average number, raise a number of risks and considerations for future research and practice planning. Focusing on the provision of more accessible and equitable palliative care for all persons, irrespective of age, must be the goal of future palliative care research and policy action.

## Figures and Tables

**Table 1:** Comparisons across younger and older subjects (*N* = 19,397).

****	Younger adults	Older adults	*P*
****	Means	Means	
Total care setting transitions	3.9	3.4	.00
Inpatient hospital discharges (count)	1.6	1.6	.63
Total inpatient days	36.2	42.7	.00
Provincial hospital stays	1.6	1.2	.00
Provincial hospital length of stays	32.4	39.9	.00
Regional hospital stays	.3	.3	.08
Regional hospital length of stays	24.9	28.4	.04
Local hospitalizations	.6	.8	.00
Local hospital stays	24.9	34.5	.00
Number of procedures	3.6	2.0	.00
Number of surgical procedures only	2.6	1.3	.00
Palliative care visits	1.3	1.2	.00
Cancer care visits	1.9	1.6	.00
Outpatient visits	6.2	4.6	.00
Outpatient procedures	12.0	8.7	.00
ER visits	3.2	2.7	.00
ER procedures	4.1	3.6	.00
Day surgery visits	2.9	2.4	.11
Day surgery procedures	17.5	17.4	.96

Mean differences were tested using the *t*-test for independent samples.

**Table 2:** Summary of logistic regression findings for younger and older subjects (*n* = 10,897; reference category—older subjects).

	Predictors	B	S.E	Wald	df	*P*	OR*
1	Total stay days	.01	.00	67.27	1	.00	1.01
2	Number of diagnoses	.03	.00	78.31	1	.00	1.03
3	Number of procedures	−.08	.02	24.52	1	.00	.92
4	Number of surgical procedures	−.09	.02	17.76	1	.00	.92
5	Number of care setting transitions	−.09	.01	69.01	1	.00	.91
6	Number of outpatient clinic visits	−.02	.00	34.46	1	.00	.98
7	Number of outpatient clinic procedures	.00	.00	2.69	1	.10	1.00
8	Number of emergency room visits	−.02	.01	15.61	1	.00	.98
9	Number of emergency room procedures	−.03	.01	18.07	1	.00	.98
	Constant	1.55	.04				

*OR stands for Odds Ratios.
